# Investigation of a thermostable multi-domain xylanase-glucuronoyl esterase enzyme from *Caldicellulosiruptor kristjanssonii* incorporating multiple carbohydrate-binding modules

**DOI:** 10.1186/s13068-020-01709-9

**Published:** 2020-04-11

**Authors:** Daniel Krska, Johan Larsbrink

**Affiliations:** 1 Division of Industrial Biotechnology, Department of Biology and Biological Engineering, Chalmers University of Technology, 412 96 Gothenburg, Sweden; 2 Wallenberg Wood Science Center, Chalmers University of Technology, 412 96 Gothenburg, Sweden

**Keywords:** Xylan, Biomass, Thermostability, Glucuronoyl esterase, Xylanase, Carbohydrate-active enzyme, Lignin–carbohydrate complexes, Carbohydrate-binding module, *Caldicellulosiruptor kristjansonii*

## Abstract

**Background:**

Efficient degradation of lignocellulosic biomass has become a major bottleneck in industrial processes which attempt to use biomass as a carbon source for the production of biofuels and materials. To make the most effective use of the source material, both the hemicellulosic as well as cellulosic parts of the biomass should be targeted, and as such both hemicellulases and cellulases are important enzymes in biorefinery processes. Using thermostable versions of these enzymes can also prove beneficial in biomass degradation, as they can be expected to act faster than mesophilic enzymes and the process can also be improved by lower viscosities at higher temperatures, as well as prevent the introduction of microbial contamination.

**Results:**

This study presents the investigation of the thermostable, dual-function xylanase-glucuronoyl esterase enzyme *Ck*Xyn10C-GE15A from the hyperthermophilic bacterium *Caldicellulosiruptor kristjanssonii*. Biochemical characterization of the enzyme was performed, including assays for establishing the melting points for the different protein domains, activity assays for the two catalytic domains, as well as binding assays for the multiple carbohydrate-binding domains present in *Ck*Xyn10C-GE15A. Although the enzyme domains are naturally linked together, when added separately to biomass, the expected boosting of the xylanase action was not seen. This lack of intramolecular synergy might suggest, together with previous data, that increased xylose release is not the main beneficial trait given by glucuronoyl esterases.

**Conclusions:**

Due to its thermostability, *Ck*Xyn10C-GE15A is a promising candidate for industrial processes, with both catalytic domains exhibiting melting temperatures over 70 °C. Of particular interest is the glucuronoyl esterase domain, as it represents the first studied thermostable enzyme displaying this activity.

## Introduction

Recent trends in industrial biofuel and biomaterial production have been focused on the use of lignocellulosic plant biomass—composed mainly of cellulose, hemicelluloses, and lignin—as a renewable feedstock to produce the desired end products [1]. Depending on the source of the plant biomass the proportion of these components vary, but typically hemicelluloses make up between 25–40% of the dry plant material in industrial crops [1–4]. In both hardwood trees and grasses, the most abundant hemicellulose is xylan, comprising between 15–50% of the cell dry weight of the plant, and approximately one-third of all renewable organic carbon on earth [1–3, 5, 6]. The xylan backbone consists of a chain of xylose residues linked in a β-1,4 configuration [1]. These backbone sugars can be appended by a variety of other sugars and non-carbohydrate moieties, leading to a multitude of different possible xylan structures [7, 8]. In many cases, backbone or other sugars are linked to lignin in so-called lignin–carbohydrate complexes (LCCs), which pose another challenge in extraction of sugars by increasing the cell wall recalcitrance to degradation [9, 10].

In addition to being a valuable and still underutilized source of sugars, the xylan polysaccharides cover and protect the cellulose fibrils from degradation, so removal of the xylan is essential if cellulose is to be degraded (e.g. for biofuel production) [5]. While pretreatment methods to remove a majority of xylans and other hemicelluloses are available, they render the hemicelluloses unsuitable for biofuel production or other downstream uses [11]. In addition, pretreatment methods can convert the hemicellulose sugars into inhibitory compounds (such as furfural), which have a negative impact on later microbial fermentations [12]. Enzymatic degradation of xylan does not generate these harmful effects and is therefore of interest both in processes focusing on cellulose and more holistic ones targeting all cell wall carbohydrates. Indeed, in many industrial applications, cellulases and xylanases are used simultaneously in order to provide the greatest yields of usable sugars [13].

Enzymatic plant biomass degradation is carried out by carbohydrate-active enzymes (CAZymes). These are grouped into classes and families in the CAZy database (http://www.cazy.org; [14]) based on their amino acid sequences, which consequently govern their structure and activity. Enzymatic degradation of xylan is typically achieved by xylanases found in glycoside hydrolase families 10 (GH10) and 11 (GH11), although xylanase activity has also been observed for members of families 5, 7, 8, 30, 43, 98, and 141 [3, 14–18]. Xylanases exist in two major categories: *endo*-acting xylanases, which randomly cleave backbone linkages in xylan, releasing xylooligosaccharides of varying lengths, and *exo*-acting xylanases, which remove d-xylose residues from the non-reducing ends of poly- or oligosaccharides [19].

As mentioned, xylan, as well as other polysaccharides, can often be found covalently bound to lignin in LCCs [9, 20]. These LCCs are highly prevalent, and the majority of lignin in hardwoods (and all lignin in softwoods) are suggested to exist in such complexes, with the attachment predominantly to the hemicelluloses in the cell wall [9, 21, 22]. LCCs add stability and recalcitrance to plant materials, and the covalent bonds between lignin and polysaccharides within the biomass impede lignin removal during pretreatment [9, 23]. It has previously been shown that the presence of LCCs, and the difficulty in removing lignin from the overall biomass, has a direct negative impact on the efficacy of commercial xylanases and cellulases in releasing mono- and disaccharides [24]. Both *endo*- and *exo*-acting xylanases can physically be blocked from acting on the xylan by the LCCs, and a portion of the carbohydrates will also remain covalently linked to lignin after the enzymatic hydrolysis. Xylan is bound to lignin in LCCs primarily through linkages to ferulic and glucuronic acid (GlcA) moieties [25, 26]. Enzymes which cleave the ferulic acid linkage in LCCs are known as feruloyl esterases (FAEs), and belong to the carbohydrate esterase family 1 (CE1) [14, 27]. While extensive data on CE1 enzymes do not exist in the literature, structural and mechanistic insights into these enzymes have however been available for around two decades [28]. Conversely, glucuronoyl esterase (GE) enzymes, known to be able to cleave the ester linkages between GlcA moieties in xylan and the aromatic alcohols on lignin, have since their discovery only recently begun to receive more attention [29–35].

To date, the only enzyme family containing GE enzymes is the carbohydrate esterase family 15 (CE15). While fungal GEs have been proposed to require 4-*O*-methylation on the GlcA moiety for activity, recent work on bacterial GEs indicate that the methylation is not essential for many enzymes [29, 32]. The GlcA–lignin ester bond is a significant contributor to the cell wall rigidity, and its cleavage can allow other carbohydrate-acting enzymes—e.g. xylanases—to reach their targets within the plant biomass. To date, all characterized GEs are either single-domain proteins, or are coupled with an N-terminal carbohydrate-binding module 1 (CBM1) domain [36]. This module is thought to allow the enzyme to bind easier to insoluble substrates, and thus increase the overall activity [37]. The majority of studied CE15 enzymes are mesophilic, with the lone exceptions being *St*GE1 and *St*GE2 from *Myceliophthora thermophila* [34, 38]. While these enzymes can be regarded as thermophilic, with temperature optima around 50–60 °C [38], they are still far below the 70–90 °C temperature optima commonly exhibited in enzymes produced by hyperthermophilic bacteria [39].

Several strategies employed by microorganisms for optimal activity of carbohydrate-acting enzymes have been described in the literature [40]. The two most commonly known strategies are either secretion of a large quantity of soluble extracellular enzymes (cellulases, hemicellulases, and other biomass-degrading enzymes), which is a common strategy in fungi, or the production of complexed enzymes in so-called cellulosomes, most commonly utilized by anaerobic microorganisms [40, 41]. Cellulosomes are large protein complexes, consisting of a non-catalytic scaffold protein, onto which multiple enzymes and carbohydrate-binding domains (CBMs) are bound through cohesin–dockerin domain interactions [42–44]. The cellulosomes allow for increased efficiency of the degradation process, due to the proximity of synergistic enzymes in the complex, and the limited diffusion of their substrates [45]. Unfortunately, while cellulosomes represent powerful systems for the degradation of plant biomass, they have proven difficult to produce solubly at industrially relevant scales [46]. In addition to the two commonly known strategies of plant biomass degradation described, a third strategy has recently been highlighted, where secreted enzymes consist of one polypeptide chain comprising multiple catalytic and carbohydrate-binding domains [47]. This strategy can be considered a midway strategy between free enzymes and cellulosome complexes, as the catalytic domains can cooperate synergistically, while still being soluble and not needing the large, complicated structural support of the cellulosome. In particular, the cellulase CelA from *Caldicellulosiruptor bescii*, with its reported striking ability to rapidly degrade crystalline cellulose has showcased the potential of such multi-catalytic enzymes [47]. These multi-catalytic enzyme architectures have been shown to be a common feature in genomes of the *Caldicellulosiruptor* genus [48, 49]. Additionally, as *Caldicellulosiruptor* bacteria are hyperthermophilic organisms thriving at temperatures around 80 °C, their enzymes are also highly thermostable. These thermostable enzymes can be very useful for industrial processes, and indeed, hyperthermophilic enzymes are currently sought after and used in industry today [50].

In the present study, we have investigated a novel enzyme, *Ck*Xyn10C-GE15A, which consists of seven individual CAZyme domains: two catalytic domains (GH10 xylanase and CE15 glucuronoyl esterase) and five CBMs. The enzyme is encoded by the anaerobic Gram-positive bacterium *Caldicellulosiruptor kristjanssonii*, originally isolated from an Icelandic hot spring [51]. *C. kristjanssonii* has an optimal growth temperature of 78 °C and is able to grow on cellulose, xylose, pectin and starch, making its enzymes an intriguing target for the study of thermophilic CAZymes [51]. In addition, the thermophilic nature of *Caldicellulosiruptor* enzymes presents several advantages for lignocellulose degradation, including easier degradation of the plant material at these high temperatures, lower risks of microbial contamination, and greater overall stability and resistance to the harsh conditions of industrial bioprocesses [52]. *Ck*Xyn10C-GE15A was biochemically characterized to determine its suitability for use in biomass degradation applications. Both the xylanase and the carbohydrate esterase catalytic domains were characterized in detail and, additionally, binding studies were carried out for the different CBMs. The results revealed how the xylanase is able to act on different xylan substrates, and the GE domain represents the first highly thermostable GE reported to date. The work also highlights the distinct binding patterns of the different CBMs, as well as their function in enhancing the thermostability of the protein.

## Results and discussion

**Fig. 1:**
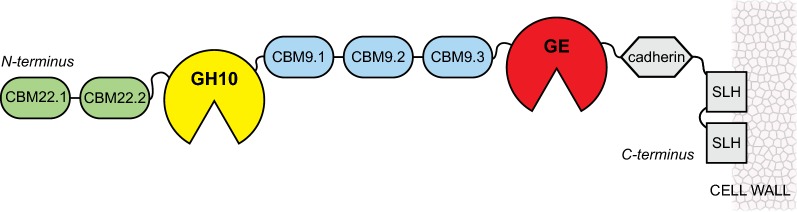
Domain architecture of *Ck*Xyn10C-GE15A outlining the seven domains investigated in this study as well as the three additional C-terminal domains that were not. The surface layer homology (SLH) domains are predicted to anchor the enzyme to the cell wall, and thereby enable both binding to substrates and catalysis in the proximity of the cell

*Ck*Xyn10C-GE15A is an enzyme containing two distinct putative catalytic modules: a GH10 xylanase and a GE from CE15. In addition, two N-terminal CBM22 domains as well as three CBM9 domains sandwiched between the catalytic domains were predicted, as well as a cadherin-like domain and two surface layer homology (SLH) domains following the GE domain at the C-terminus (Fig. 1). The GH10 xylanase domain, *Ck*Xyn10C, is 91% identical on the amino acid level to the previously characterized *exo*-xylanase Calkro_0402 from *C. kronotskyensis*, and the CE15 GE domain, *Ck*GE15A, is 39% identical on the amino acid level to the previously characterized C-terminal domain of CesA from *Ruminococcus flavenfaciens*, as determined using the protein–protein basic local alignment search tool (BLAST) algorithm [53, 54]. The two catalytic domains of *Ck*Xyn10C-GE15A can reasonably be expected to act in a synergistic manner on xylan-containing biomass, considering their predicted activities and fusion into a single enzyme. The unusually large number of CBMs (five) found in this protein can be expected to contribute towards activity or stability in some way, such as preference in binding different substrates, or simply to compensate for the reduced binding affinities typically observed at higher temperatures.

In order to study the function, thermostability and possible synergy between the domains of *Ck*Xyn10C-GE15A, we chose to first study the individual domains’ interactions with model substrates, followed by investigation on their potential to depolymerize xylan in complex biomass. As the enzyme is encoded by a hyperthermophilic organism, we further investigated the effect of temperature on enzyme activity and stability. Despite extensive efforts to crystallize and conduct structural studies on these domains, crystal structures were not possible to obtain using multiple commercially available crystal screens. The CBM domains were studied with respect to their stabilizing effect on the catalytic domains, as well as their individual binding capabilities to insoluble substrates. The full-length enzyme, comprising the fused catalytic domains and carbohydrate-binding modules (but excluding the cadherin and SLH domains), unfortunately proved impossible to produce despite extensive efforts.

### Determination of protein thermal stability

Melting temperature analysis revealed a temperature of unfolding between 40 °C and 80 °C for each subunit tested (Table 1). Interestingly, the tested CBM9 domains had the lowest melting temperatures of all the domains, with T_m_ values under 50 °C. However, the melting profile for each of these CBM9 domains showed an extremely broad peak (data not shown), suggesting that the unfolding is a slow process, and that it is possible that these domains retain some structure or function at temperatures higher than their observed melting points.

**Table 1:** Melting temperatures for various *Ck*Xyn10C-GE15A domains, as determined using a Thermofluor assay

Domain	Temperature (°C)
CBM22.2	62.6 ± 1.8
*Ck*Xyn10C	ND
CBM22-CBM22-*Ck*Xyn10C	78.6 ± 0.1
CBM9.1	ND
CBM9.2	49.1 ± 3.1
CBM9.3	42.6 ± 1.8
*Ck*GE15A	72.0 ± 0.2

The results are presented as the average of triplicate experiments with standard errors

*ND* unable to be determined using this assay

### Determination of glucuronoyl esterase activity

Measurement of the GE activity at both 22 °C and 40 °C was conducted by a continuous assay developed recently [32], using the substrates benzyl-d-glucuronoate (BnzGlcA), allyl-d-glucuronoate (AllGlcA), and methyl-d-glucuronoate (MeGlcA; Table 2). No activity could be detected on the substrate methyl-d-galacturonate, indicating that *Ck*GE15A has a strict preference for GlcA-derived esters, in contrast to several other bacterial enzymes able to also act on galacturonic acid-derived ester substrates [32]. The pH optimum of *Ck*GE15A was determined to be 7.0, using BnzGlcA as substrate. However, for more accurate comparisons with other characterized CE15 enzymes in literature, assays were conducted at pH 7.5. At 22 °C, the *K*_*m*_ of *Ck*GE15A for BnzGlcA was relatively high compared to previously characterized bacterial GEs [32], at 18.5 ± 0.7 mM (Table 2). For the smaller substrates AllGlcA and MeGlcA, the reactions were not possible to saturate, suggesting much higher *K*_*m*_ values. The *k*_cat_/*K*_*m*_ values for AllGlcA and MeGlcA were 5- and 40-fold lower than for the BnzGlcA substrate, respectively. The results are consistent with previously reported activities for multiple CE15 enzymes [36], and support the notion that GE enzymes generally have a preference for large side groups attached to their glucuronic acid substrates, in keeping with their proposed role to target the ester bonds between xylan polysaccharides and the lignin network. This is also reflected in the fact that the active sites of GE enzymes are surface-exposed and in addition to xylan- or xylooligosaccharide binding sites have large surfaces where lignin fragments could be accommodated [32, 55, 56]. In fact, a recent study showed how GE enzymes may bind longer xylooligosaccharides, where previously only monosaccharides had been successfully modeled as ligands in protein 3-D structures [57].

**Table 2:** Kinetic parameters of *Ck*GE15A on various synthetic substrates

Condition	Temperature (°C)	*K*_*m*_ (mM)	*k*_*cat*_ (1/min)	*k*_*cat*_/*K*_*m*_ (s^−1^M^−1^)
Benzyl-d-glucuronoate	22	18.5 ± 0.7	1.18 ± 0.04	1.06 ± 0.05
Allyl-d-glucuronoate	22	Cannot be saturated up to 40 mM		0.21 ± 3.2×10^−3^
Methyl-d-glucuronoate	22	Cannot be saturated up to 40 mM		0.028 ± 4.6×10^−4^
Benzyl-d-glucuronoate	40	8.6 ± 0.9	3.39 ± 0.14	6.56 ± 0.38

The results are presented as the average of triplicate experiments with standard errors

The reaction of the *Ck*GE15A domain with the BnzGlcA substrate at 40 °C showed an approximately twofold reduction in *K*_*m*_ and a threefold increase in *k*_cat_. It was not possible to conduct reactions at higher temperatures due to rapid autohydrolysis of the substrates that resulted in extremely high background, which shows that the model substrates are not thermostable, as reported previously [58]. Therefore, an optimal temperature for this domain could not be determined. However, based on the different reaction rates at 40 °C compared to 22 °C, it is likely that *K*_*m*_ would continue to decrease and the *k*_cat_ increase until the temperature optimum for the enzyme is reached, which is likely to be considerably closer to the enzyme’s melting temperature of 72 °C, and the enzyme represents the most thermostable GE reported to date. When compared to previously characterized bacterial GE enzymes, *Ck*GE15A appears to have significantly weaker activity than most [32]. The *k*_cat_/*K*_*m*_ value for *Ck*GE15A was up to three orders of magnitude lower than that reported for the most active bacterial CE15 enzymes, and in the low range for bacterial CE15 enzymes overall [32]. At 40 °C, the individual *k*_cat_ and *K*_*m*_ values were however comparable to several previously studied bacterial enzymes, such as *Sl*CE15A from *Spirosoma linguale* and *Ot*CE15B from *Opitutus terrae* [32]. As we were unable to assay the activity of *Ck*GE15A at elevated temperatures due to the temperature sensitivity of the substrates, this comparison is likely not a good reflection of the enzyme’s true catalytic efficiency at high temperatures. Additionally, *Caldicellulosiruptor* bacteria have been shown to glycosylate CAZymes [59, 60], and possibly also *Ck*Xyn10C-GE15A is glycosylated in vivo and requires these post-translational modifications for full activity, as it has previously been shown that glycosylation is of great importance to the activity of other esterases, increasing their reaction rates by several orders of magnitude [61]. All of these factors considered, it is likely that the true kinetic parameters for *Ck*GE15A is significantly higher than reported here.

No previously discovered or characterized GE enzyme is linked to CBM9 domains, as studied GE enzymes have been either single-domain proteins or linked to N-terminal CBM1 domains [36]. It is possible that the CBM9 modules provide a different function than previously seen in CE15-linked CBMs. Both CBM1 and CBM9 proteins are thought to primarily have cellulose-binding functions, though previous work has shown that CBM9 modules associated with xylanases can also bind insoluble xylan, although in that case the CBM9 still displayed a greater affinity for cellulose than for xylan [14, 54, 62]. As there are no bacterial CBM1 modules, it is possible that the CBM9 modules present in *Ck*Xyn10C-GE15A are performing a similar function as the CBM1 modules would in fungal enzymes [14].

Interestingly, there does not seem to be another GE enzyme in any published *Caldicellulosiruptor* genome. Close homologs of many CAZymes present within the *Caldicellulosiruptor* genus exist in multiple organisms, and often as multiple copies within the same organism. Many multi-domain enzymes in this genus arise through domain shuffling, leading to similar protein arrangements within the different species [63]. Indeed, the *Ck*Xyn10C domain, as well as the arrangement of CBMs surrounding it appears to be present in multiple *Caldicellulosiruptor* genomes. The presence of the GE in *C. kristjanssonii*, and also within a multi-domain enzyme, could indicate a unique evolutionary adaptation by this organism to environmental conditions not encountered by other *Caldicellulosiruptor* organisms.

### Xylanase activity investigation

**Fig. 2:**
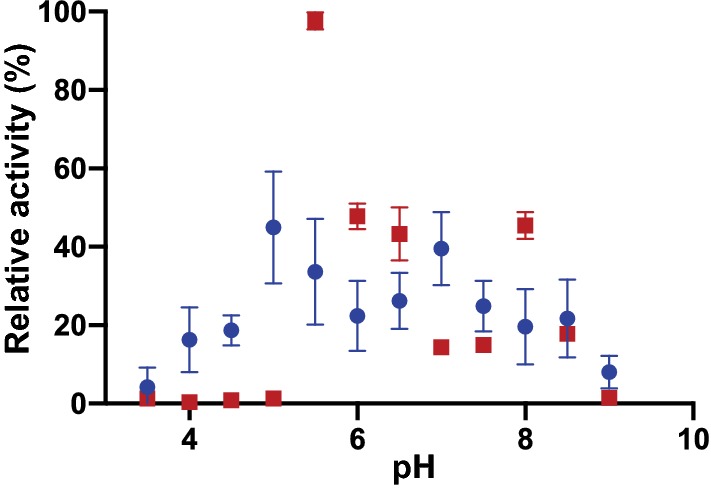
Relative activity of the GH10 domain (red square) and the GH10 domain with both N-terminal CBM22 domains (blue circle) over a range of different pHs. Sodium acetate buffer was used up to pH 5.5, sodium phosphate from pH 6 to 8, and Tris for pH 8.5 and 9. The results are presented as the average of triplicate experiments with standard errors

Xylanase activity for both the *Ck*Xyn10C domain on its own, and the same domain coupled to the two N-terminal CBM22 domains was measured by monitoring the release of reducing sugars during hydrolysis of different xylan polysaccharides (Tables 3, 4, Additional file 1: Figure S2). We attempted to also produce a protein with only the second CBM22 domain and the *Ck*Xyn10C domain, unfortunately, this construct did not express in *E. coli*. The *Ck*Xyn10C domain by itself displayed optimal activity at pH 5.5, using wheat arabinoxylan as a substrate (Fig. 2). However, activity was also assayed at pH 7 to correspond with the pH dependence of the *Ck*GE15A domain. Interestingly, *Ck*Xyn10C showed dual pH optima when combined with the N-terminal CBM22 domains, as it was also 64% active compared to the optimum at pH 7.5 (Fig. 2). Possibly, the observed pH optimum at 5.5 for the *Ck*Xyn10C domain is an experimental artifact, as similar pH optima of both the xylanase and GE domains would be plausible, and more suitable for the environment where *C. kristjanssonii* grows. *Ck*Xyn10C-GE15A is predicted to be found extracellularly due to both a signal peptide sequence located at the N-terminus of the protein, and the C-terminal SLH domains, which are typically found anchored to the cell wall outside the cell (Fig. 1). Compared to the most similar described GH10 domain to *Ck*Xyn10C, Calkro_0402 protein from *C. kronotskyensis* as mentioned above, Calkro_0402 differs from *Ck*Xyn10C by not exhibiting a dual pH optimum. Calkro_0402 is instead maximally active at pH 5.5 and displays a significant activity decrease at pH 7. Calkro_0402 is not found fused to a CE15 domain, and it might be possible that the xylanase domain in the *C. kristjanssonii* enzyme over time has adapted to better complement the GE domain in order to achieve maximum synergistic effects [54], in keeping with the observation that almost all GEs characterized to date have exhibited neutral-to-basic pH optima [31, 32, 57].

**Table 3:** Kinetic parameters for *Ck*Xyn10C, assays run using 50 nM GH10 for 5 min

Condition	pH 7		pH 5.5	
	*K*_*m*_ (g/L)	*k*_cat_ (1/s)	*K*_*m*_ (g/L)	*k*_cat_ (1/s)
Birchwood xylan, 40 °C	ND	ND	N/A	N/A
Birchwood xylan, 65 °C	6.2 ± 0.8	80 ± 4.8	5.9 ± 1.1	173 ± 14.4
Beechwood xylan, 40 °C	14.6 ± 4.8	110 ± 22.1	N/A	N/A
Beechwood xylan, 65 °C	5.1 ± 0.7	173 ± 10.7	2.6 ± 0.3	387 ± 13.6
Wheat arabinoxylan, 65 °C	56.3 ± 21.7	1940 ± 627	10.0 ± 1.4	960 ± 74.2

The results are presented as the average of triplicate experiments with standard errors

*ND* not determined due to low activity, *N/A* not assayed

**Table 4:** Kinetic parameters for CBM22-CBM22-*Ck*Xyn10C, assays run using 500 nM CBM-CBM22-*Ck*Xyn10C for 5 min

Condition	pH 7		pH 5.5	
	*K*_*m*_ (g/L)	*k*_cat_ (1/s)	*K*_*m*_ (g/L)	*k*_cat_ (1/s)
Birchwood xylan, 40 °C	20.4 ± 2.9	34 ± 3.2	N/A	N/A
Birchwood xylan, 65 °C	9.6 ± 0.5	81 ± 2.4	N/A	N/A
Birchwood xylan, 80 °C	6.6 ± 0.4	80 ± 2	10.2 ± 1	366 ± 24
Beechwood xylan, 40 °C	11.5 ± 1.5	23 ± 1.7	N/A	N/A
Beechwood xylan, 65 °C	9.6 ± 0.6	77 ± 2.7	N/A	N/A
Beechwood xylan, 80 °C	10.5 ± 0.7	110 ± 4.2	8.4 ± 1.4	321 ± 17
Wheat arabinoxylan, 80 °C (50 nM enzyme)	8.4 ± 1.2	633 ± 4.5	13.2 ± 2	1953 ± 18

The results are presented as the average of triplicate experiments with standard errors

*N/A* not assayed

**Fig. 3:**
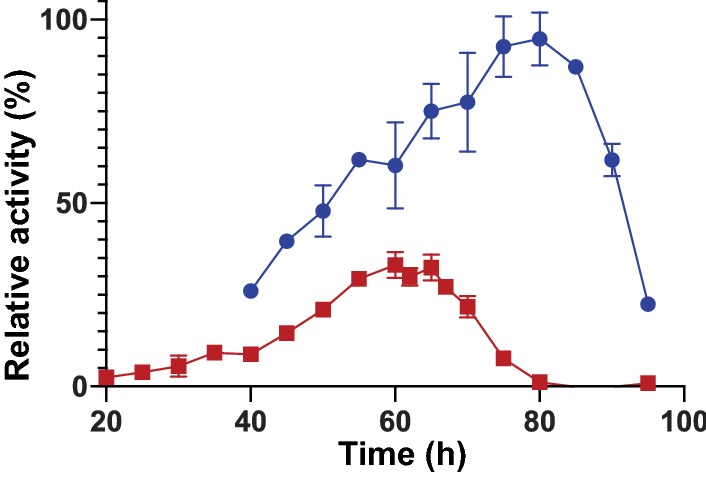
Activity of CBM22-CBM22-*Ck*Xyn10C (500 nM; blue) and the single *Ck*Xyn10C domain (50 nM; red) as an effect of temperature on the beechwood xylan substrate. A clear increase in both activity and thermostability can be observed when the CBM22 domains are attached to the catalytic xylanase domain. The results are presented as the average of triplicate experiments with standard errors

The temperature required for optimal activity of the *Ck*Xyn10C domain by itself was 65 °C (Fig. 3). With the addition of the two N-terminal CBM22 domains, the temperature optimum was increased significantly, to 80 °C, which is comparable with the optimal growth temperature of *C. kristjanssonii* (78 °C). This thermostabilizing effect of CBM22 modules has been seen in several other xylanase enzymes, and is as such not entirely surprising [64, 65]. Interestingly, despite the stabilization by the CBMs, the GH10 domain was approximately as efficient on its own at 65 °C compared to the variant coupled to the family 22 CBMs at 80 °C (Tables 3, 4). This could suggest that the N-terminal CBMs are in some way interfering with the enzymatic function of *Ck*Xyn10C in this three-domain configuration, as higher reaction temperatures should in theory lead to faster reactions. This could be caused by the CBMs partially blocking the active site, as they are located on flexible linkers within the protein and would be able to move around in solution. Considering the environment in which *C. kristjanssonii* is found, it is possible that this could be a trade-off for the enzyme to be stable as well as functional in vivo. Kinetic parameters for the *Ck*Xyn10C enzyme without adjoining CBMs could not be performed at 80 °C, as the temperature profile demonstrated almost no activity at that temperature (Fig. 3).

Due to the structural similarities of birch- and beechwood xylan (glucuronoxylan), the difference seen in enzyme activity when acting on the two different substrates was somewhat surprising (Table 3). The difference between the two substrates was sufficiently great that activity on birchwood xylan was not possible to be determine at 40 °C. However, previous work by Liu et al. has shown at least one xylanase, also from the *Caldicellulosiruptor* genus, which displays an increased specific activity on beechwood xylan compared to birchwood xylan [66]. The *k*_cat_ values also reflect this difference in substrate preference, with those for beechwood being approximately twofold higher than those for birchwood for the singular *Ck*Xyn10C domain. Interestingly, this difference disappears for the CBM22-CBM22-GH10 protein, where the catalytic efficiency on beech and birch xylan is nearly identical (Table 4). Although the *k*_cat_ values are nearly identical for birchwood and beechwood xylan for the CBM22-CBM22-GH10 construct, there are significant differences in the *K*_*m*_ values for these different substrates. The decrease in apparent substrate specificity on birchwood xylan suggests that the substitution pattern on the xylan backbone is different between the two polysaccharides, and that at high substrate concentrations the enzyme is still able to find sufficient accessible sites in the polysaccharide to reach full activity. In the case of both the *Ck*Xyn10C domain on its own, as well as linked to the two N-terminal CBMs, *k*_cat_ values proved to be much higher at pH 5.5 than at pH 7 (Tables 3, 4).

We next measured the activity of both the single xylanase domain, as well as the domain linked to both CBM22 domains on wheat arabinoxylan. Compared to the activities observed on the hardwood xylans, above, the enzyme variants showed a greatly increased activity on the arabinoxylan polysaccharide (Tables 3, 4). For *Ck*Xyn10C, a tenfold higher *k*_cat_ was observed at pH 5.5 and 65 °C compared to the results on beechwood xylan, and similarly a sixfold higher *k*_cat_ was observed for the CBM22-CBM22-GH10 construct. In contrast to the assays on hardwood xylans, above, the *Ck*Xyn10C domain displayed a twofold higher *k*_cat_ value on wheat arabinoxylan at pH 7 compared to pH 5.5, though the *K*_*m*_ at pH 7 was also fivefold higher, which resulted in a higher specificity constant at pH 5.5 (Table 3).

As wheat arabinoxylan differs from hardwood xylans in having arabinofuranosyl substitutions and a generally denser substitution pattern [67, 68], the results suggest a possible preference for more highly decorated xylan chains for the *Ck*Xyn10C xylanase domain. Another explanation of the results could be that while wheat arabinoxylan is fully soluble at the concentrations tested, the hardwood xylans are mostly insoluble, and therefore the xylanase might be apparently more active due to substrate accessibility.

### Carbohydrate-binding module thermostability and binding to polysaccharides

**Fig. 4:**
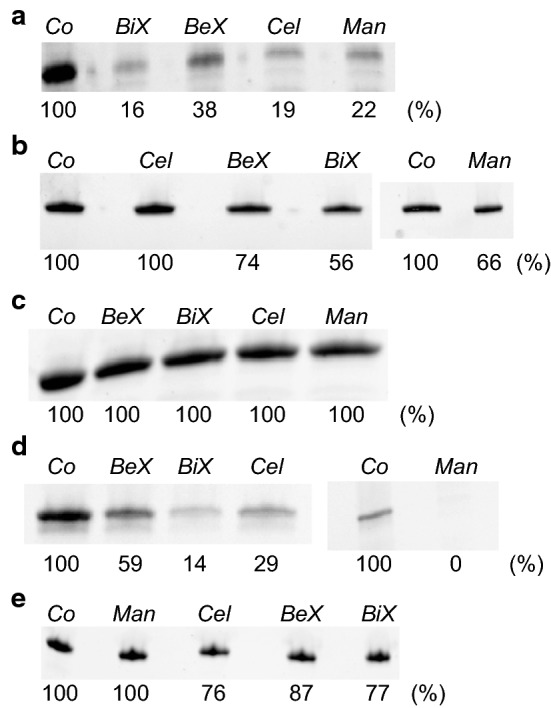
Binding of CBM22.1-CBM22.2 (**a**), CBM22.2 (**b**), CBM9.1 (**c**), CBM9.2 (**d**), and CBM 9.3 (**e**). Differences in binding no substrate (Co), birch xylan (BiX), beech xylan (BeX), cellulose (Cel), and mannan (Man) are seen by comparing the relative intensity of the bands, determined using the Image Lab software

As mentioned, *Ck*Xyn10C-GE15A contains five different CBMs: two N-terminal CBMs belonging to family 22 and three ‘internal’ CBMs from family 9 found between *Ck*Xyn10C and *Ck*GE15A (Fig. 1). Going from the N-terminus, the domains were named CBM22.1, CBM22.2, CBM9.1, CBM9.2, and CBM9.3, respectively. The domains were assayed for their ability to bind insoluble polysaccharides in pull-down studies (Table 5, Fig. 4).

**Table 5:** Binding of CBMs to various insoluble substrates, as determined by a pull-down assays

CBM	Beech xylan	Birch xylan	Mannan	Cellulose
22.1–22.2	++	++	++	++
22.2	+	++	++	–
9.1	–	–	–	–
9.2	++	++	+++	++
9.3	+	+	–	+

Minus symbols signify no binding. Plus symbols represent binding, with the number of symbols increasing for stronger apparent binding

CBM22.1 proved to be unstable after recombinant production and purification, and could therefore not be studied as a single protein. Instead, a stable fusion protein comprising both CBM22.1 and CBM22.2 was designed and produced, and its differences compared to CBM22.2 alone allowed some indications on the function of the CBM22.1 domain. CBM22.2 was produced as a stable, singular protein, and the pull-down studies showed it was able to bind beech xylan, birch xylan and ivory nut mannan, but not cellulose. The dual CBM22 protein was able to, similar to CBM22.2, bind to both beech and birch xylan, as well as mannan. Additionally, the dual construct was able to bind to cellulose, and in general exhibited a stronger binding to the polysaccharides compared to the single CBM22.2. The results indicate that CBM22.1 could be a cellulose-binding domain, possibly also able to bind to xylans and mannan, while the function of the CBM22.2 is likely to bind hemicelluloses, similar to previously described CBM22 proteins [65].

All of the CBM9 proteins were possible to produce as single soluble proteins, though, unexpectedly, CBM9.1 had to be targeted to the periplasm to yield viable *E. coli* cells during production. CBM9.1 did not show binding to any of the tested polysaccharides, but might possibly bind carbohydrates not assayed in our experiments, or act merely as a spacer domain in the full-length enzyme. In contrast, CBM9.2 bound all of the tested insoluble polysaccharides. It appeared to bind birch xylan much more effectively than beech xylan, on which it showed the least binding ability out of any of the polysaccharides. Additionally, it bound mannan extremely effectively, as no detectable protein could be observed left in the soluble fraction. CBM9.3 was able to bind beech and birch xylan, as well as cellulose, but appeared to be the least effective at binding these insoluble substrates of any of the tested CBMs. No binding of CBM9.3 to mannan could be observed, suggesting an inability to bind carbohydrates exhibiting axial OH-moieties at the C-2 position of the sugar ring.

Previously, it has been suggested that CBM22 modules lacking conserved substrate-binding residues are responsible for thermostabilizing effects when linked to xylanases [64]. Sequence analysis of the two CBM22 modules of *Ck*Xyn10C-GE15A shows that CBM22.2 does not appear to lack conserved binding residues. CBM22.1 contains three of five previously identified “essential” binding residues, however, the full set of five residues are present in fewer than half of the members of the family and whether these are essential or not is currently not clear [69]. Our results indicate that these CBM22 domains may instead serve a dual function in both thermostabilizing the GH10 domain as well as assisting in substrate binding. While we cannot conclude that CBM22.1 has a carbohydrate-binding ability due to the instability of the single domain, CBM22.2 shows definite substrate binding and the dual CBM22 construct has a different binding ability compared to the single CBM22.2. These results collectively support the hypothesis of a dual binding and stabilizing functionality of the CBM22s.

### Evaluation of enzyme synergy in biomass hydrolysis

**Fig. 5:**
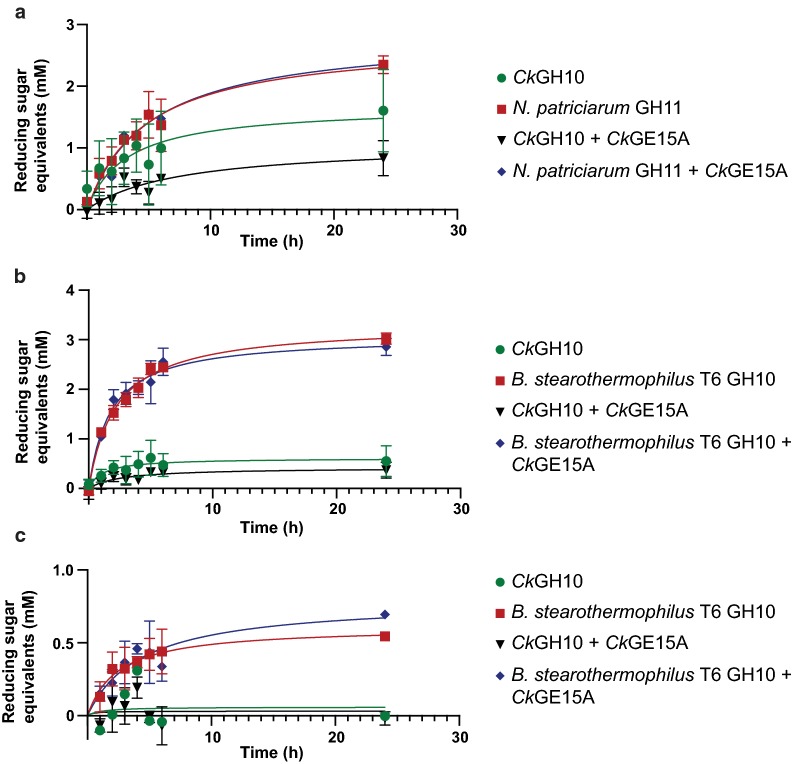
Assays on complex biomass using the *C. kristjansonii* catalytic domains and commercial xylanases in various combinations. Corn cob biomass at 30 °C (**a**) and 60 °C (**b**), and wheat straw at 60 °C (**c**). No activity could be detected on the wheat straw at 30 °C, and the *Ck*GE15A enzyme on its own resulted in no release of detectable sugars in any of the conditions. The results are presented as the average of triplicate experiments with standard errors

The activities and fusion of *Ck*GE15A and *Ck*Xyn10C into a single enzyme suggest cooperativity between the domains, where the glucuronoyl esterase would aid the xylanase by detaching xylan polysaccharides from lignin. The effect of adding *Ck*GE15A in combination with a xylanase (either purchased from a commercial source, or *Ck*Xyn10C) was investigated on both milled wheat straw and corn cob biomass. Assays were completed using equimolar amounts of xylanase and *Ck*GE15A, in order to best mimic the natural, linked form of the enzyme. *Ck*GH10A was able to release reducing sugars from the biomass sources, to similar or lower extents compared to a commercial GH10 xylanase from *Bacillus stearothermophilus* T6 and a GH11 xylanase from *Neocallimastix patriciarum* (Fig. 5). Curiously, the enzyme was unable to release sugars from milled wheat straw despite its highest activity on the wheat arabinoxylan compared to glucuronoxylan (Tables 3, 4), suggesting that it needs to act in concert with other enzymes to be able to access the xylan chains in complex biomass. No significant difference was seen in the samples with or without *Ck*GE15A when compared to the equivalent control sample on either biomass at either temperature. Unfortunately, due to the difficulty in producing the full-length enzyme, testing the domains as linked together was not possible. The results were unexpected, as it could reasonably be hypothesized that there is a benefit to having the two catalytic domains physically linked (i.e. synergistic effects of having both acting in close physical proximity on the same biomass). It might however be possible that a physical connection between the catalytic domains is required to see boosting effects, as has been demonstrated recently for enzymes comprising fused carbohydrate esterase domains, where the added efficiency displayed by full-length enzymes was suggested to stem from the simultaneous action of both domains on their different respective substrates in close proximity [70]. Also, in a previous study investigating GE-mediated boosting of biomass hydrolysis, only a small effect on xylose release was shown, where out of the three tested enzymes, xylose release was only increased by approximately one-third for the best-performing enzyme and for the least performing GE no significant increased xylose release was observed [32]. Additionally, this study utilized GE enzymes with several orders of magnitude greater activity on synthetic substrates than was displayed by *Ck*GE15A. The current data suggest that the enhancement of xylose release by the addition of *Ck*GE15A or GEs in general is limited at best, though a full-length version of *Ck*GE15A might exhibit the expected synergy on biomass where the xylanase domain could act in concert with the GE on recalcitrant LCCs. Given the complexity of the cell wall network and macroscopic changes during hydrolysis, it is possible that the xylanase domain simply does not frequently enough encounter the sites targeted by the GE domain, or that additional enzymes need to participate in a more holistic degradation of the plant cell wall. Despite the lack of additional xylose release, GEs have previously been shown to aid in the release of several other monosaccharides, and are still considered promising for industrial applications.

## Conclusion

In this work we have shown that *Ck*Xyn10C-GE15A from *C. kristjanssonii* acts as both a xylanase and a glucuronoyl esterase, due to its multi-domain architecture. Both enzyme domains become more active as temperature increases, although the optimal temperature of the GE domain could not be determined due to substrate instability. The GE domain appears to prefer bulkier substrates, in line with its expected natural substrates in the plant cell wall, while the GH10 domain prefers arabinoxylan to glucuronoxylan in our experiments. Possibly, as the enzyme was able to act on the heavily substituted glucuronoarabinoxylan found in corn biomass, this type of xylan might prove to be the optimal substrate for the xylanase. Unfortunately, corn xylan as a pure substrate was not accessible, and such a hypothesis might be tested in future studies. We have here characterized the individual domains of *Ck*Xyn10C-GE15A, though the complete multi-domain enzyme could not be obtained due to difficulties in production and purification of the full-length 191-kDa protein. Future studies may be able to develop new approaches for the production of such large full-length enzymes. For example, full-length *Caldicellulosiruptor* proteins which have proven to be difficult to produce in *E. coli* have previously been successfully produced in *Bacillus megaterium*, which might make production in that host a viable future option [71]. A lack of boosting seen when mixing the two enzymes in the same reaction was surprising, though the results match what has previously been seen in other studies with GE enzymes. It is also possible that these enzymes would show a greater boosting effect if linked together to allow the xylanase to directly act on xylan chains liberated from LCCs. As a final note, this study also presents the first thermostable GE enzyme, which may prove useful as an inclusion in thermostable biomass-degrading enzyme cocktails.

## Methods

### Cloning

DNA sequences encoding the various domains of the *C. kristjanssonii Ck*Xyn10C-GE15A enzyme were amplified from genomic DNA (Strain ID 12137, DSMZ) using the primers listed in Additional file 1: Table S1. The gene fragments were inserted into a modified pET-28a plasmid containing a Tobacco Etch Virus protease restriction enzyme site, generously provided by Dr. N. Koropatkin, University of Michigan Medical School, using the In Fusion cloning kit (Takara Life Sciences). The plasmids were transformed into chemically competent *E. coli* Stellar cells and plated on lysogeny broth (LB) agar plates containing 30 µg/mL kanamycin and incubated overnight at 37 °C. Colonies were grown in 5 mL of LB media containing kanamycin overnight at 37 °C, and plasmid DNA was extracted using the GeneJet Plasmid Miniprep Kit (Thermo Scientific) and sequenced by Eurofins Genomics.

### Expression and protein purification

*E. coli* BL21 cells transformed with the appropriate plasmid were grown overnight in 50 mL of LB media containing 30 μg/mL kanamycin. 10 mL of overnight culture was used to inoculate 1 L of LB media. The culture was grown at 37 °C to an OD_600_ of 0.6 when protein production was induced by addition of isopropyl β-d-1-thiogalactopyranoside (Saveen and Werner) to a final concentration of 1 mM. The cells were grown for an additional 4 h at 37 °C before harvesting by centrifugation, followed by resuspension in a buffer consisting of 50 mM tris(hydroxymethyl)aminomethane (Tris), pH 8.0 and 100 mM NaCl. Cells were lysed via sonication, centrifuged, and the supernatant was taken for further purification.

Protein purification was performed using an ÄKTA FPLC (GE Healthcare) by Ni^2+^ immobilized metal affinity chromatography (IMAC), using a binding buffer containing 50 mM Tris, pH 8.0, and 100 mM NaCl, and elution buffer with the same composition as well as 250 mM imidazole. Further purification was performed by gel filtration chromatography when protein purity was insufficient, using a HiLoad Superdex 200 16/60 column (GE Healthcare). In this case, the same binding buffer as for IMAC was used.

The sequence coding for the first CBM9 in the protein sequence (from the N-terminus; CBM9.1) could not be produced using the modified pET-28a plasmids. CBM9.1 was instead cloned into a pET-22b vector, containing a PelB leader sequence targeting produced proteins to the *E. coli* periplasm, and transformed into *E. coli* Stellar cells as described above and plated on LB agar containing 100 µg/mL ampicillin. Cultures for protein production were grown as described above, though using ampicillin in place of kanamycin. Protein extraction from the periplasm was performed by osmotic shock as described previously [72]. Briefly, cells were suspended in 100 mL of 30 mM Tris pH 8, 20% w/v sucrose, and 1 mM ethylenediaminetetraacetic acid (EDTA), and incubated at 4 °C for 1 h. Cells were then centrifuged, the supernatant collected, and the pellet resuspended in 5 mM MgSO_4_ at 4 °C for an additional hour. The supernatant was again collected and combined with the previous one, and then purified by IMAC as described for other proteins above.

The second (middle) CBM9 domain (CBM9.2) was purified cytosolically as described above for the other proteins, but resulted in insoluble inclusion bodies. After sonication and centrifugation, the remaining pellet was resuspended in 8 M urea, incubated at room temperature for 20 min, centrifuged again, and the resulting supernatant was used for protein purification as described above to enable on-column re-folding.

### Determination of glucuronoyl esterase activity

Activity was measured using a spectrophotometric assay described previously [58]. Briefly, the synthetic substrate analogs benzyl-, allyl-, or methyl-d-glucuronoate or methyl-d-galacturonoate (Carbosynth) were dissolved in dimethylsulfoxide (DMSO). Release of uronic acids were determined spectrophotometrically using a FLUOstar Omega plate reader, in continuous assays using the coupled enzymatic assay K-URONIC kit (Megazyme), to monitor the NADH produced at λ=340 nm.

### Determination of xylanase activity

In order to measure the release of reducing sugars by the GH10 domain, 3,5-dinitrosalicylic acid (DNSA) assays were performed. Various concentrations of xylan from birchwood (Megazyme) or beechwood (Apollo Scientific) were incubated with 50 nM GH10 domain (500 nM for the CBM22-CBM22-GH10 construct) in 50 mM sodium phosphate buffer, pH 7 at either 40 °C, 65 °C, or 80 °C. After 5 min, the reaction was stopped by the addition of an equal volume of a solution consisting of 1% DNSA, 1% sodium hydroxide, and 0.2% phenol. The mixture was then boiled for 10 min, centrifuged briefly, transferred to a 96-well plate, and absorbance was measured on a FLUOStar Omega plate reader at 540 nm. A standard curve with varying concentrations of glucose was used to convert absorbance values to concentration of reducing sugars released. Attempts to repeat the assay with commercially purchased corn xylan were unsuccessful, as the material proved to be short oligomers rather than xylan chains [57].

### *T*_*m*_ determination

Melting temperature determination of proteins was performed using the thermofluor assay [73]. Briefly, the protein was diluted to 200–1000 nM, and mixed in a 1:20 ratio with 5× SYPRO Orange dye (Sigma-Aldrich). This was transferred to a 96-well PCR plate, sealed, and measured on a Stratagene Mx3005P Q-PCR machine (Agilent), using 492 nm/516 nm excitation/emission filters. The temperature was increased by 1 °C/min, and the change in fluorescence recorded. Data were analyzed using the TSA-CRAFT thermal shift analysis software [74].

### Enzymatic hydrolysis of biomass

The ability of the GE domain in boosting enzymatic hydrolysis of biomass was investigated on both ball-milled corn cob and ball-milled wheat straw biomass. 5% biomass was incubated for 24 h at either 30 °C or 60 °C, with combinations of *Ck*Xyn10C, *Ck*GE15A, or a commercial xylanase (*endo*-1,4-β-xylanase from *Neocallimastix patriciarum* (Megazyme) at 30 °C, and *endo*-1,4-β-xylanase from *Bacillus stearothermophilus* T6 (Megazyme) at 60 °C). Incubations without added enzyme served as controls. Samples were taken every hour for the first 6 h, and then after 24 h. A small amount of the reaction mixture, including insoluble biomass, was removed from the reaction tube and filtered through a 96-well filter plate (Millipore) to remove the insoluble biomass components. Samples were then immediately frozen in liquid nitrogen and stored until thawed and analyzed using the DNSA assay described for determination of xylanase activity.

### Substrate binding

Pull-down assays were performed by incubating 0.2 mg of CBM in 200 μL of a suspension containing 0.625 mg of insoluble polysaccharide, either beech or birch xylan, cellulose (Sigma Aldrich), or ivory nut mannan (Megazyme). The polysaccharide–CBM suspension was incubated with shaking at 2000 rpm at 40 °C for 1 h on a ThermoMixer C (Eppendorf). The samples were then centrifuged, and the supernatant collected and analyzed by SDS-PAGE by comparing band intensity with an unbound protein control (Image Lab 6.0, Bio-Rad).

## Supplementary information

**Additional file 1.** Additional table and figures.

## Data Availability

The datasets used and/or analyzed during the current study are available from the corresponding author on reasonable request

## Abbreviations

AllGlcA: Allyl-d-glucuronoate
BLAST: Basic local alignment search tool
BnzGlcA: Benzyl-d-glucuronoate
CAZy: Carbohydrate-active enzymes database
CAZyme: Carbohydrate-active enzyme
CBM: Carbohydrate-binding module
CBM1: Carbohydrate-binding module family 1
CBM9: Carbohydrate-binding module family 9
CBM22: Carbohydrate-binding module family 22
CE1: Carbohydrate esterase family 1
CE15: Carbohydrate esterase family 15
DNSA: 3,5-Dinitrosalicylic acid (assay)
FAE: Feruloyl esterase/ferulic acid esterase
GE: Glucuronoyl esterase
GH10: Glycoside hydrolase family 10
GH11: Glycoside hydrolase family 11
GlcA: Glucuronic acid
LCC: Lignin–carbohydrate complex
MeGlcA: Methyl-d-glucuronoate
SLH: Surface layer homology domain
