# Supplementary material for: Investigation of a thermostable multi-domain xylanase-glucuronoyl esterase enzyme from Caldicellulosiruptor kristjanssonii incorporating multiple carbohydrate-binding modules
Source: Biotechnol Biofuels. 2020 Apr 11;13:68. doi: 10.1186/s13068-020-01709-9 (PMC7151638; doi:10.1186/s13068-020-01709-9)
Supplement: Supplementary file 1 — Additional file 1. Additional table and figures. [file 13068_2020_1709_MOESM1_ESM.docx]

**Additional File 1** – Investigation of a thermostable multi-domain xylanase-glucuronoyl esterase enzyme from *Caldicellulosiruptor kristjanssonii* incorporating multiple carbohydrate-binding modules; **Authors:** Daniel Krska and Johan Larsbrink

**Table S1:** List of primer sequences used and the domain sequences amplified with them.

| **Domain(s)** | **Forward Primer** | **Reverse Primer** |
| --- | --- | --- |
| CBM22.1-CBM22.2-GH10-CBM9.1-CBM9.2-CBM9.3-CE15 | ACTTCCAGGGCCATAAAACGCAAGGTACACCGACGATCTT | TGCTCGAGTGCGGCCTAATCAGCGTTTCGGTAAAGTGTGGGC |
| CBM22.1 | ACTTCCAGGGCCATAAAACTCAAGGTACACCGACAATTTTAA | TGCTCGAGTGCGGCCTAGTTCGACGGGACACATACCAGAA |
| CBM22.1-CBM22.2 | ACTTCCAGGGCCATAAAACTCAAGGTACACCGACAATTTTAA | TGCTCGAGTGCGGCCACTAGTCGAAATCAAGGGCTGGTGA |
| CBM22.1-CBM22.2-GH10 | ACTTCCAGGGCCATAAAACGCAAGGTACACCGACGATCTT | TGCTCGAGTGCGGCCTATACTGATGGTTCCACAATCGCCCAGT |
| CBM22.2 | ACTTCCAGGGCCATAAGTCGCTCGAATTCTACTTGGACAA | TGCTCGAGTGCGGCCACTAGTCGAAATCAAGGGCTGGTGA |
| GH10 | ACTTCCAGGGCCATATCGACTTTGACTTTTACCTTGACGACTTC | TGCTCGAGTGCGGCCTATACTGATGGTTCCACAATCGCCCAGT |
| CBM9.1 | GAATTAATTCGGATCTGATTGACGGAGTAATGGACAGAGAATACAAAG | GTGCGGCCGCAAGCTGCTTGACACTGTCCGCCATG |
| CBM9.2 | ACTTCCAGGGCCATATCGCTACGGTATATAAAGGGACGCC | TGCTCGAGTGCGGCCTACGGTCCTACCAGCTCAATACACC |
| CBM9.3 | ACTTCCAGGGCCATAAGAGGATCTATGTAAGCTACGGTTCGCC | TGCTCGAGTGCGGCCTAGGGGTTGATTGGTACGACAGCAT |
| CE15 | ACTTCCAGGGCCATATAGAGACCTTGCCTGACTCGTTCACC | TGCTCGAGTGCGGCCTAATCAGCGTTTCGGTAAAGTGTGGGC |


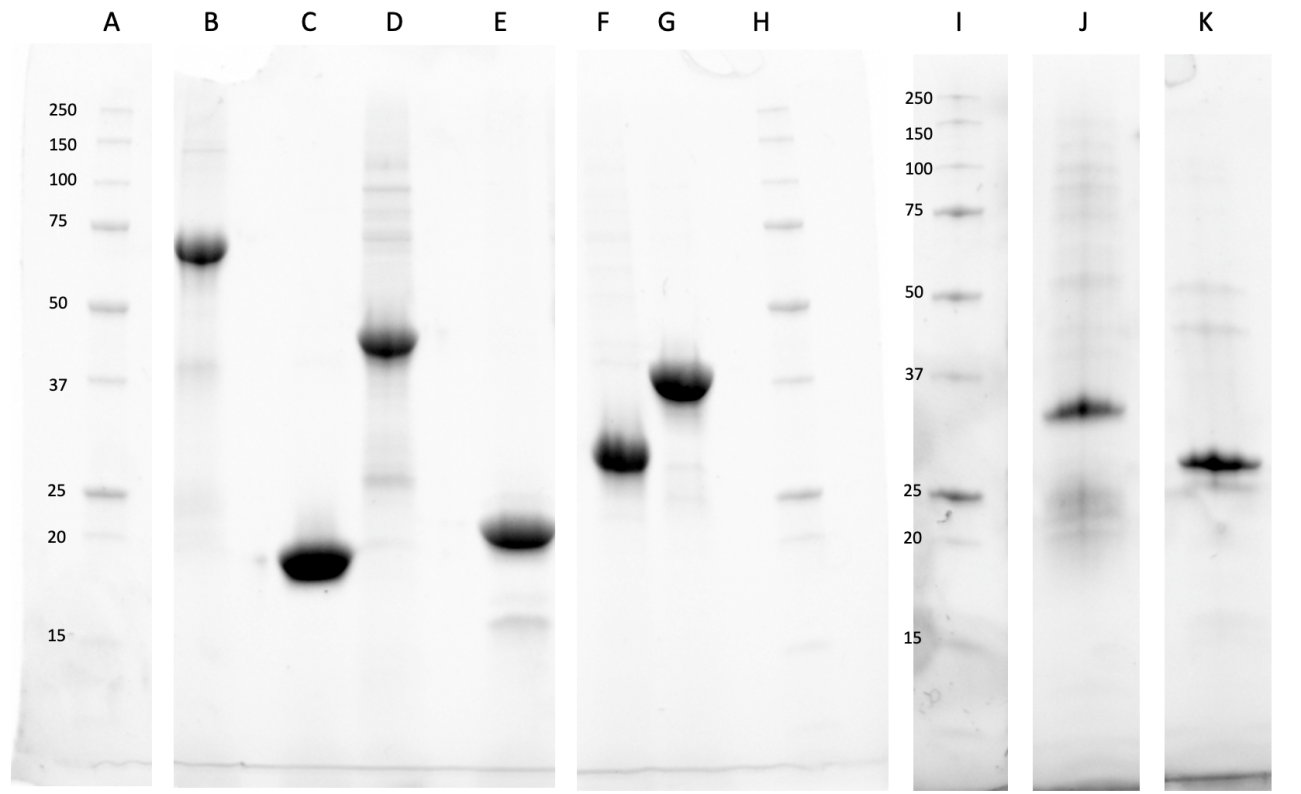


**Figure S1:** SDS-PAGE gel visualizations of the purified proteins used in this study. From left to right: molecular weight marker (A), CBM22.1-CBM22.2-CkXyn10C (B), CBM22.2 (C), CkXyn10C (D), CBM9.1 (E), CBM9.3 (F), CkGE15A (G), molecular weight marker (H), molecular weight marker (I), CBM22.1-CBM22.2 (J), CBM9.2 (K). Lanes I-K are all from the same gel, with unrelated lanes removed for clarity.


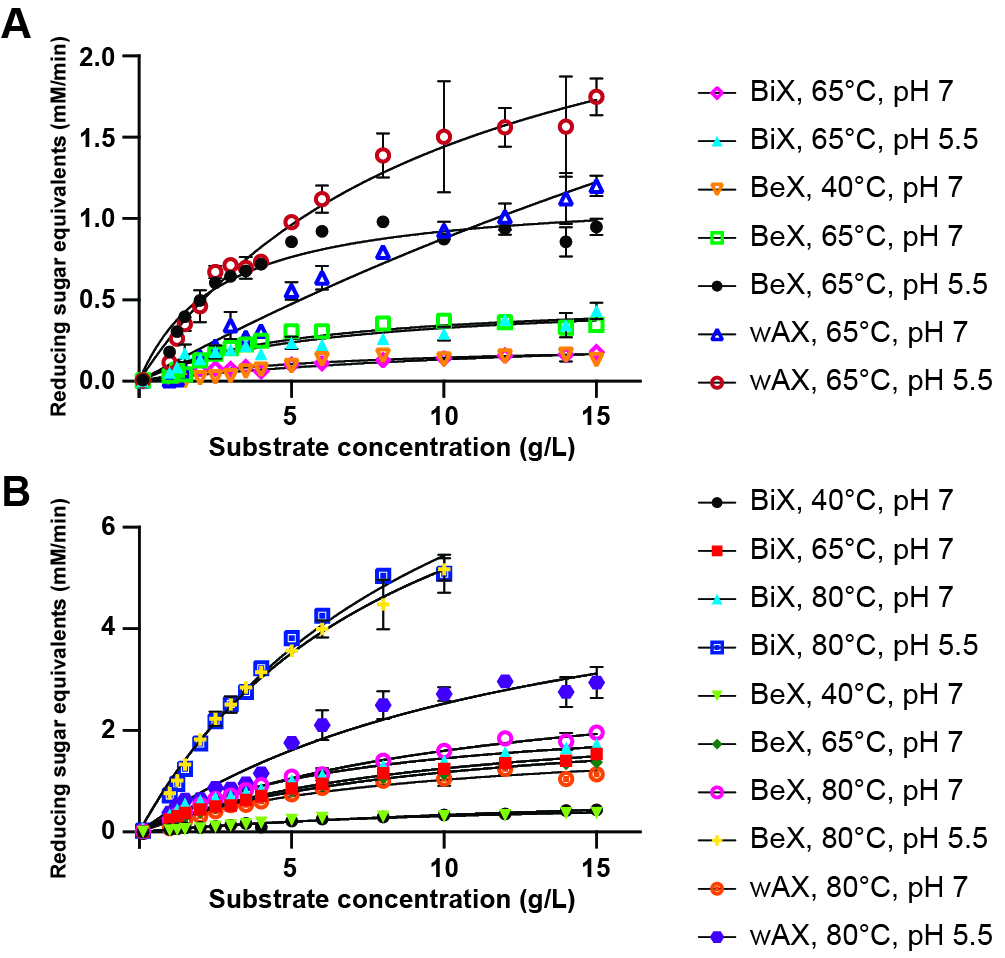


**Figure S2:** Activity measurements of the single *Ck*Xyn10C GH10 domain (A) and the CBM22.1-CBM22.2-*Ck*Xyn10C enzyme (B). Various substrates were tested at different temperatures (birchwood xylan – BiX, beechwood xylan – BeX, wheat arabinoxylan - wAX), and at both pH 5.5 and pH 7.
